# Rhizonin A from *Burkholderia* sp. KCTC11096 and Its Growth Promoting Role in Lettuce Seed Germination

**DOI:** 10.3390/molecules17077980

**Published:** 2012-07-03

**Authors:** Sang-Mo Kang, Abdul Latif Khan, Javid Hussain, Liaqat Ali, Muhammad Kamran, Muhammad Waqas, In-Jung Lee

**Affiliations:** 1 School of Applied Biosciences, Kyungpook National University, Daegu 702-701, Korea; Email: kmoya@hanmail.net (S.-M.K.); latifepm78@yahoo.co.uk (A.L.K.); m.kamran60@gmail.com (M.K.); agronomist89@gmail.com (M.W.); 2 Department of Botany, Kohat University of Science and Technology, Kohat 26000, Pakistan; 3 Department of Biological Sciences and Chemistry, College of Arts and Sciences, University of Nizwa, Nizwa 33, Oman; Email: javidhej@unizwa.edu.com (J.H.); malikhejric@gmail.com (L.A.); 4 Department of Chemistry, Kohat University of Science and Technology, Kohat 26000, Pakistan

**Keywords:** *Burkholderia* sp. KCTC11096, rhizonin A, growth promotion, lettuce seed

## Abstract

We isolated and identified a gibberellin-producing *Burkholderia* sp. KCTC 11096 from agricultural field soils. The culture filtrate of plant growth promoting rhizobacteria (PGPR) significantly increased the germination and growth of lettuce and Chinese cabbage seeds. The ethyl acetate extract of the PGPR culture showed significantly higher rate of lettuce seed germination and growth as compared to the distilled water treated control. The ethyl acetate fraction of the *Burkholderia* sp. was subjected to bioassay-guided isolation and we obtained for the first time from a *Burkholderia* sp. the plant growth promoting compound rhizonin A (**1**), which was characterized through NMR and MS techniques. Application of various concentrations of **1** significantly promoted the lettuce seed germination as compared to control.

## 1. Introduction

Secondary metabolites have mainly been isolated and characterized from microbes for industrial and medicinal purposes [1,2]. For the microbiologist studying ecology, it is apparent that secondary metabolites play a role *in vivo* and are important for various metabolic interactions between microorganisms and their plant hosts. Natural products continue to be an important source of new pharmaceutical products [2,3]. It has been shown that the: (1) secondary metabolite synthesis by rhizobacteria may correspond to its respective taxon and ecological niche while (2) metabolic interactions may enhance the synthesis of several metabolites as well as (3) effect plant growth and development [3,4]. Thus, rhizobacteria are one of such sources which can be used for intelligent screening of bioactive metabolites. The biological activities of bacterial strains may vary with the biotope from which they are isolated [5]. Similarly, it is imperative to consider the niche from where rhizobacteria have been isolated as it will determine the diversity of biochemical potential of the rhizobacteria [3,4,5]. The isolated metabolites of bacteria belong to diverse classes of compounds including steroids, xanthones, phenols, isocoumarins, perylene derivatives, quinones, furandiones, terpenoids, depsipeptides, and cytochalasines [5]. The biotechnological use of these metabolites for pharmaceutical or agrochemical products is still in the developmental stage. 

To increase agricultural efficiency, crop plant growth promotion by using eco-friendly alternatives is essential for sustainable agricultural production. Thus, the use of natural processes to improve the quantity and quality of agronomics can result in development of expanded food production system, which will ultimately bring sustainability to the ecological systems [6]. In the present research work, we isolated a Plant Growth-Promoting Rhizobacteria (PGPR) strain *Burkholderia* sp. KCTC11096 from agricultural soils. Earlier to this, the PGPR had significantly higher rate of phosphate solubilization potential during growth in its culture medium, and the PGPR produced biologically active gibberellins and various organic acids [7,8]. Furthermore, its application to various other crop plants like cucumber and tomato has significantly increased the plant biomass and growth [9]. In present study, we aimed to isolate and characterize the plant growth-promoting compound(s) responsible for this activity using column chromatographic techniques, HPLC, nuclear magnetic resonance (NMR) and MS analysis.

## 2. Results and Discussion

**Figure 1:**
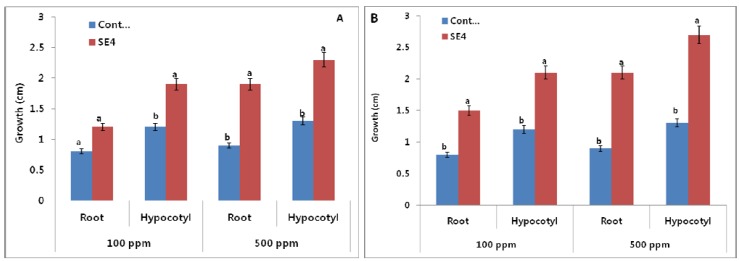
Effect of culture filtrate on the root and hypocotyls of (**A**) lettuce and (**B**) Chinese cabbage seeds using two different concentrations 100 and 500 ppm. SE4 refers to *Burkholderia* sp. (*n* = 10). For each set of treatment, the different letter indicates significant differences (*P <* *0.05*) between SE4 and control treatments as evaluated by Duncan multiple range test. Error bars refers to SE.

The *Burkholderia* sp. KCTC 11096BP (SE4) was grown in nutrient broth for seven days on 25 °C at 120 rpm. The culture filtrate (CF) of *Burkholderia* sp. was applied to lettuce and Chinese cabbage seeds which were incubated for three days at room temperature in the dark. The control seeds were treated with bacteria-free nutrient broth and distilled water using the same concentrations. The results of the initial screening assay showed that the CF of *Burkholderia* sp. enhanced the seed germination of lettuce and Chinese cabbage seeds and significantly increased the root and hypocotyls length of lettuce seeds as compared control treatments (Figure 1). We used two different concentrations *i.e.*, 100 and 500 ppm which increased the root and hypocotyls length in a dose dependent manner. The effect of lowest concentration (100 ppm) on the growth of root of lettuce seeds was not significantly different between control and SE4, while at maximum concentration there was significant difference (Figure 1).

**Figure 2:**
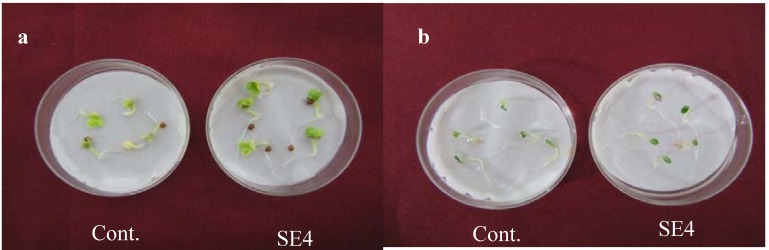
Effect of ethyl acetate extract (500 ppm) on the growth of (**a**) lettuce and (**b**) chinese cabbage seeds.

The CF of *Burkholderia* sp. was successively partitioned with chloroform and ethyl acetate and the extracts were vacuum dried and bioassayed using lettuce and Chinese cabbage seeds. Two different concentrations (100 and 500 ppm) of the two extracts were assessed for their effects on seed germination. The results showed that the ethyl acetate extract was significant in its effects, not only in the germination of the lettuce and Chinese cabbage seeds, but also by improving the root and hypocotyls length of the seeds (Figure 2). The effect was more stimulatory on Chinese cabbage than lettuce seeds. The effect was growth promoting as compared to control. The CF (5 liter) of *Burkholderia* sp. was grown and successively partitioned with ethyl acetate to yield 2.1 g of a yellowish gummy extract.

**Figure 3:**
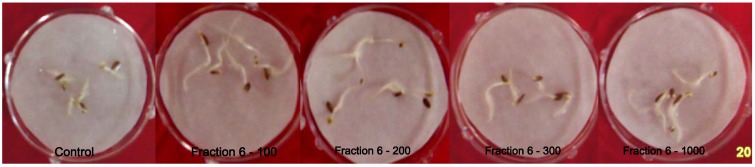
Effect of various concentrations (100, 200, 300 and 1,000 ppm) of fraction 6.

The bioactive ethyl acetate extract was chromatographed on a C18 column into six different fractions (see Experimental). All the six fractions were bioassayed for their effect on the germination and growth of the lettuce seeds. We used a concentration gradient of 100, 200, 300 and 1,000 ppm of the extract in 5% DMSO. The results showed that the extract of fraction number F-6 (elution of 100% ethyl acetate) showed significant stimulatory effects as compared to other fractions as well as the control lettuce seeds (Figure 3). All the concentrations (100, 200, 300 and 1,000 ppm) were significant in promoting the seed germination and growth root and hypocotyles of lettuce seeds as compared to control.

**Figure 4:**
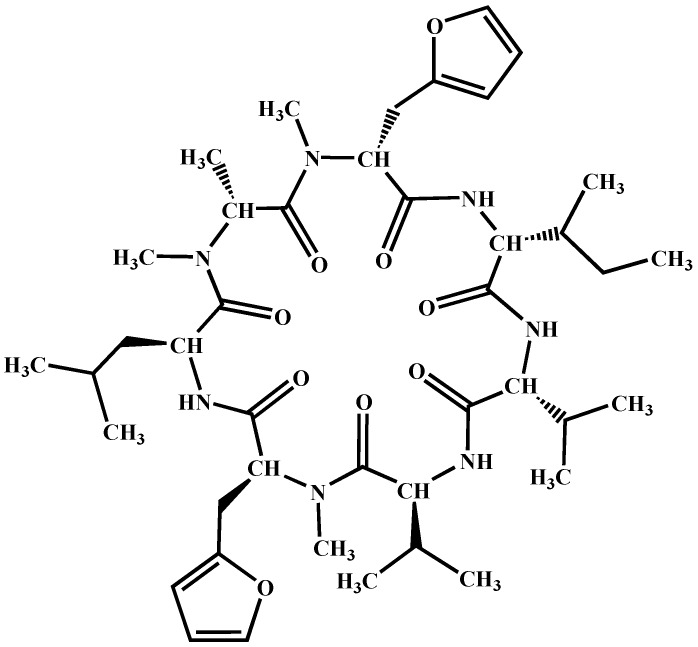
Structure of compound **1**.

The fraction F-6 was subjected to high performance column chromatography (HPLC) to further purify the bioactive constituents. The HPLC analysis showed three prominent peaks which were isolated and again bioassayed. The result showed that F-6-1 has sighnificantly higher lettuce seed germination activity as compared to control and other fractions. The F-6-1 was identified as a peptide and was characterized with the help of NMR and MS techniques. The MS revealed the molecular mass and hence the molecular formula of **1** along with some major fragments of the peptide skeleton. The heptapeptide skeleton with three *N*-CH_3_ groups was further supported by ^1^H-NMR and ^13^C-NMR spectroscopic techniques. The ^1^H-NMR spectrum of compound **1** exhibited three singlets at δ 3.11, 3.15 and 3.23, which indicated the presence of *N*-CH_3_ groups in the molecule. The resonances for the NH and CH protons were observed in the regions δ 7.08–7.03 and δ 4.29–3.81 respectively. These observations were also confirmed through ^13^C-NMR spectrum, which clearly indicated the presence of seven carbonyl signals at δ 172.8, 172.6, 170.9, 169.7, 168.9, 167.6, and 166.9, three *N*-CH_3_ signals at δ 61.5, 61.3, and 60.3, seven signals for *α-*methyl carbons at δ 57.9–45.9, and the aromatic signals for the two furan moieties in the range δ 137.4–116.2. All these observations coupled with the literature search proved the structure as Rhizonin A (**1**). This compound (Figure 4) was previously reported by Partida-Martinez *et al.* [10], Steyn *et al.* [11] and Wilson *et al.* [12].

**Figure 5:**
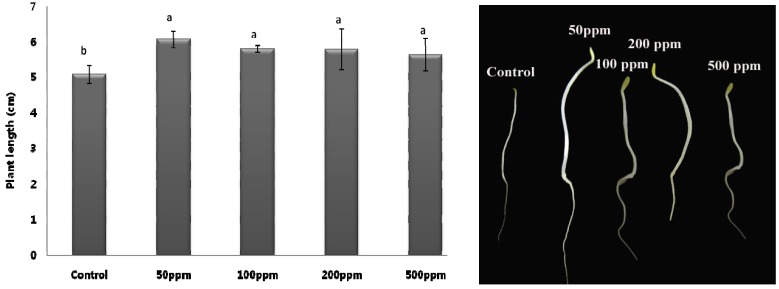
Effect of compound (**1**) on the germination and growth of lettuce seeds using various concentrations (*n* = 5). For each set of treatment, the different letter indicates significant differences (*P* < 0.05) between SE4 and control treatments as evaluated by Duncan multiple range test. Error bars refers to SE.

The effect of compound **1** on the seed germination and growth of lettuce seeds was assessed. Four different concentrations of 50, 100, 200 and 500 ppm were used for the bioassays. The application of 50 ppm showed a significant stimulatory effect towards the germination of lettuce seeds. At other concentrations (100, 200 and 500 ppm), though, the plant length was reduced as compared to 50 ppm but it was higher than that of the DW applied control seedlings (Figure 5).

Rhizonin A (**1**) is a non-ribosomal cyclic heptapeptide that was ﬁrst isolated in 1983 from the fungus *Rhizopus microsporus* which had feasted on *Mozambican* groundnuts [10,11,12]. The compound was reported as the ﬁrst mycotoxins from lower fungi, that is, a fungus belonging to the order Mucorales of the class Zygomycetes. After two decades, however, the true producer of **1** was revealed to be not the fungus, but rather the endo-fungal bacteria *Burkholderia rhizoxina* [10,13]. Biological investigations have showed that **1** is a potent and non-specific hepatotoxin in rats, though damage to the liver can be evoked by many chemical substances. While the administration of a high dose of **1** in animals such as rats and ducklings induced death, **1** strongly suppressed an increase in over the long term. However, it has been unclear whether this effect was a side effect of a potent hepatotoxic effect or a potential direct effect on body weight [10,11,12].

We have isolated a plant growth promoting molecule, which was previously found to be a hepatotoxin in animals. Previously, various studies have shown that PGPRs can produce plant growth promoting substances like gibberellins, auxin and salicylic acid, *etc.* [1,14,15]. We also noted that the *Burkholderia* sp. produce gibberellins GA_1_, GA_3_, GA_4_, GA_9_, GA_12_, GA_15_, GA_20_, and GA_24_ and promoted the cucumber plants [7,9]. In present study, compound **1** isolated from *Burkholderia* sp. also promoted the growth of both Chinese cabbage and lettuce seed. The results suggest that compound **1** might have influenced GAs biosynthesis pathways and thus promoted not only the seed germination but also increased their lengths as compared to control. Previously, there are a number of studies available that bioactive secondary metabolites can influence endogenous GAs biosynthesis to germinate the seed early [16]. A similar effect was observed for the smoke-derived bioactive butanliode and karrikins [16]. We also presume that the compound is of peptide origin, that’s why creating a promoting effect as compared to other chemical substances offering inhibitory effects. This is the first study on this compound while it needs further studies.

## 3. Experimental

### 3.1. General

The ^1^H- and ^13^C-NMR spectra were recorded in CD_3_OD using TMS as internal standard on a Bruker spectrometer (AVANCE III 500, Madison, WI, USA) operating at 500 MHz. The chemical shift values are reported in ppm (*δ* ) units and coupling constants (*J*) in Hz. For GC/MS an Agilent 7890A-5975C with MSD (Santa Clara, CA, USA) equipped with JMS-HX-110 with data acquisition system and JMS-DA 500 mass spectrometer was used. High performance liquid chromatography was performed on a Shimadzu CBM-10 (Tokyo, Japan) instrument with UV/V dectector-SPD-10A, refractive index detector—RID-10A, LC-10A, which was equipped with a reversed-phase column (Luna C18 100A, 4.6 × 250 mm, Phenomenex, Torrance, CA, USA). Methanol, ethyl acetate and Milli-Q water were used as elution solvents in chromatography. 

### 3.2. Isolation and Screening

Soil samples were collected from Geongbuk Province (Andong, Chungju, Jinju, Sangju and Yecheon) and 10 g of soil was transferred to 250 mL flasks containing 100 mL of sterile Amies solution [17]. Resulting suspensions were serially diluted (10^−4^) and 0.1 mL aliquots were plated on tryptic soy agar (TSA; Merck Co., Darmstadt, Germany), yeast extract-malt agar (YMB; Merck Co.), potato dextrose agar (PDB; Merck Co.) for isolation of microbes. Bacterial cultures were incubated for 24 h at 30 °C for seven days. The bacterial colonies differentiated by their morphology, pigmentation and growth rate were selected, counted and re-streaked on fresh TSA medium. For long term preservation, bacteria were stored in 50% glycerol at −20 °C. SE4 (*Burkholderia* sp.) was selected as previously we found that it also produce gibberellins in its culture and improve the growth attributes of other crop plants [9]. Beside, its ability to produce GAs, we were interested to assess its bioactive secondary metabolites potentials. 

### 3.3. PGPR Culture Conditions and Growth

PGPR strain, SE4 was grown initially in nutrient broth (100 mL) for 7 days at 26 °C at 120 rpm. The culture filtrate of PGPR was screened on lettuce (*Lactuca sativa*) and Chinese cabbage (sp name) seeds. Upon significant results, SE4 was selected for further analysis and studies. The nutrient broth containing PGPR was transferred to 5 liter of nutrient broth. The broth was kept for 30 days in shaking incubator (30 °C, 200 rpm). After incubation, the supernatant and bacterial pallets were separated through centrifugation (at 5,000 *g* at 4 °C for 15 min). The supernatant was subjected to bioassay-guided isolation.

### 3.4. Extraction and Isolation

The CF was assayed for the promoting effect on the lettuce and Chinese cabbage seeds. Upon positive bioactive results of the PGPR, the filtrated supernatant was further extracted with an equal volume of ethyl acetate (EtOAc) three times to obtain an extract. The EtOAc extract of the CF of the strain was checked for their germination effects on both the seeds. SE4 showed significant activity and the 30 days grown 5 liter culture was extracted with EtOAc three times to obtain a crude gummy extract (1.2 g). The extract was chromatographed on a 15 g Davisil C18 column (90–130 um; Alltech, Deerﬁeld, IL, USA) using 60:30 MeOH in water, 100% MeOH, 60:30 MeOH in EtOAc and 100% EtOAc eluent. All the 6 fractions were bioassayed for their germination against both the indicator seeds. Fraction 6 (100% EtOAc) was found bioactive in the germination of lettuce seeds. Fraction 6 was further chromatographed on HPLC using the following conditions:

(1). Shimadzu CBM-10 coupled with UV-VIS detector (SPD-10A) having pump A and B (LC-10AD).
(2). Solvent A—100% MeOH; Solvent B—Water with 5% acetic acid.
(3). Solvent program: 0–20 min (50% = A; 50% = B); 20–40 min (80% A; 20% B); 40–60 min (100% A; 0% B).
(4). Flow rate was 1 min/mL.
(5). C18 Column (Luna 5 µm; 100A; 250× 4.60 mm).
(6). 20 µL of single injection having a 100 mg of fraction.

### 3.5. Characterization and Identification

After HPLC isolation, compound **1** was isolated from the Fraction 6. The structures of the compound was elucidated by using ^1^H-NMR and ^13^C-NMR while the mass was identified using GC/MS. The NMR and GC/MS data was compared with the literature and we found that it corresponded to a known compound reported previously by Partida-Martinez *et al.* [10]. ^1^H-NMR: δ 7.08 (s, 1H), 7.07 (s, 1H), 7.06–7.04 (m, 2H), 7.03 (s, 1H), 6.83 (d, *J* = 8.1 Hz, 2H), 6.51 (d, *J* = 8.1 Hz, 2H), 4.29–3.81 (m, 7H), 3.23 (s, 3H), 3.15 (s, 3H), 3.11 (s, 3H), 3.41–2.79 (m, 15H), 2.26–1.57 (m, 21H), 1.44 (d, *J* = 6.8 Hz, 3H), 1.39 (d, *J* = 6.6 Hz, 3H), 1.35 (d, *J* = 6.6 Hz, 3H), 1.29 (d, *J* = 6.5 Hz, 3H), 1.12 (t, *J* = 6.1 Hz, 3H), 1.07 (d, *J* = 5.8 Hz, 3H), 0.85 (t, *J* = 7.0,6.8 Hz, 3H); ^13^C-NMR: δ 172.8, 172.6, 170.9, 169.7, 168.9, 167.6, 166.9, 137.4, 132.1, 131.3, 131.1, 129.7, 129.5, 128.1, 127.7, 116.2, 61.5, 61.3, 60.3, 57.9, 57.7, 54.7, 48.9, 48.7, 46.5, 45.9, 39.4, 37.7, 37.1, 29.9, 29.8, 29.6, 25.8, 23.7, 23.5, 22.8, 22.2, 21.4, 18.9, 16.7, 15.5, 12.6.

### 3.6. Bioassay

Two different concentrations of 100 ppm and 500 ppm of culture filtrate and extract of each fraction were prepared by dissolving it in 2.5% DMSO or DW. Initial concentration was 1,000 ppm. Glass dish of 27 mm diameter with a lid and a filter paper (27 mm ø, Type Roshi Kaisha, Ltd, Tokyo, Japan) was used in glass dish. The dilutions were subjected on the filter paper and thus allowed to spread over it. Five lettuce or Chinese cabbage seeds were placed on it and the dishes were sealed and packed for incubation for 72 hours at room temperature. The control has only DW or 2.5% DMSO solution. The experiment was repeated thrice.

### 3.7. Statistical Analysis

The significant differences among the mean values of various treatments were determined using Duncan’s multiple range tests (DMRT) at 95% *CI* using Statistic Analysis System (SAS 9.1, Cary, NC, USA).

## 4. Conclusions

Plant growth promoting rhizobacteria are known for their growth regulatory role during the plant life cycle. However, few strains have been elucidated for their role in production of bioactive secondary metabolites and crop plant growth. We isolated and characterized the plant growth promoting compound rhizonin A (**1**) from a *Burkholderia* sp. through a bioassay guided method. **The** application **of gibberellins produced by** *Burkholderia* sp. has previously promoted the shoot growth of cucumber plants; while in present study compound **1** enhanced the seed germination and growth of lettuce and Chinese cabbage seeds. The application of such PGPR at the field level might result in early seed germination and plant growth which can be helpful to increase crop productivity.
